# New function of aldoxime dehydratase: Redox catalysis and the formation of an expected product

**DOI:** 10.1371/journal.pone.0175846

**Published:** 2017-04-14

**Authors:** Masatoshi Yamada, Yoshiteru Hashimoto, Takuto Kumano, Seiya Tsujimura, Michihiko Kobayashi

**Affiliations:** 1 Institute of Applied Biochemistry and Graduate School of Life and Environmental Sciences, The University of Tsukuba, Tsukuba, Ibaraki, Japan; 2 Division of Materials Science, Faculty of Pure and Applied Sciences, The University of Tsukuba, Tsukuba, Ibaraki, Japan; Universidade Nova de Lisboa Instituto de Tecnologia Quimica e Biologica, PORTUGAL

## Abstract

In general, hemoproteins are capable of catalyzing redox reactions. Aldoxime dehydratase (OxdA), which is a unique heme-containing enzyme, catalyzes the dehydration of aldoximes to the corresponding nitriles. Its reaction is a rare example of heme directly activating an organic substrate, unlike the utilization of H_2_O_2_ or O_2_ as a mediator of catalysis by other heme-containing enzymes. While it is unknown whether OxdA catalyzes redox reactions or not, we here for the first time detected catalase activity (which is one of the redox activities) of wild-type OxdA, OxdA(WT). Furthermore, we constructed a His320 → Asp mutant of OxdA [OxdA(H320D)], and found it exhibits catalase activity. Determination of the kinetic parameters of OxdA(WT) and OxdA(H320D) revealed that their *K*_m_ values for H_2_O_2_ were similar to each other, but the *k*_cat_ value of OxdA(H320D) was 30 times higher than that of OxdA(WT). Next, we examined another redox activity and found it was the peroxidase activity of OxdAs. While both OxdA(WT) and OxdA(H320D) showed the activity, the activity of OxdA(H320D) was dozens of times higher than that of OxdA(WT). These findings demonstrated that the H320D mutation enhances the peroxidase activity of OxdA. OxdAs (WT and H320D) were found to catalyze another redox reaction, a peroxygenase reaction. During this reaction of OxdA(H320D) with 1-methoxynaphthalene as a substrate, surprisingly, the reaction mixture changed to a color different from that with OxdA(WT), which was due to the known product, Russig’s blue. We purified and identified the new product as 1-methoxy-2-naphthalenol, which has never been reported as a product of the peroxygenase reaction, to the best of our knowledge. These findings indicated that the H320D mutation not only enhanced redox activities, but also significantly altered the hydroxylation site of the substrate.

## Introduction

We have widely investigated the microbial metabolism of toxic compounds containing a triple bond between carbon and nitrogen (e.g., nitriles [1–4] and isonitriles [5–7]). Microbial nitrile degradation proceeds through two different enzymatic pathways [8–9]. The enzymes (e.g., nitrilase [10–12], nitrile hydratase (NHase) [13–16], and amidase [17–19]) that are involved in both pathways have received much attention in applied [8, 20] as well as academic fields [12, 21]. From the *Pseudomonas chlororaphis* B23 [22] strain, we isolated an interesting heme enzyme [aliphatic aldoxime dehydratase (OxdA)] that catalyzes the dehydration of aliphatic aldoximes [R—CH = N—OH] to the corresponding nitriles. In this strain, the OxdA-dependent nitrile synthetic step is coupled with the nitrile-degradative step involving amidase and NHase [23, 24], which was formerly used as an industrial catalyst for acrylamide manufacture [8, 20, 25]. Through determination of the crystal structure of OxdA, and enzymological and physicochemical characterization, including spectroscopic analysis of the wild-type and mutant OxdAs [26–33], we clarified the full picture of the aldoxime dehydratase reaction mechanism, which is very unique and intriguing.

Among hemoproteins, myoglobin (Mb), which does not catalyze redox reactions primarily, has been reported to have the ability for the redox reactions [34–38]. However, such ability of aldoxime dehydratases (including OxdA) has never been reported previously. In the present study, we found that OxdA has three redox activities (catalase, peroxidase and peroxygenase). In the catalase reaction [39] and the chloroperoxidase reaction [40], an Asp residue located at the distal heme site of a mutant Mb [41] has been suggested to cause enhancement of the formation of a ferryl porphyrin cation radical (compound I) as a reaction intermediate [42, 43]. We investigated the three redox activities of two OxdA mutants in which the His320 corresponding to the Asp residue was mutated. (i) OxdA(H320D) (His320 being converted to Asp) was constructed to enhance the redox activities. (ii) OxdA (H320A) (H320 being converted to Ala), which has already been identified to exhibit no aldoxime dehydratase activity, was used to examine the effect of removal of charge of the 320th residue. All redox activities of OxdA(H320D) were higher than those of the wild-type OxdA and OxdA(H320A). Furthermore, the OxdA(H320D) yielded a new reaction product during the peroxygenase reaction.

## Materials and methods

### Materials

Standard chemicals were obtained from Tokyo Kasei Kogyo Co., Ltd. (Tokyo, Japan), Wako Pure Chemical Industries Co., Ltd. (Osaka, Japan), Sigma-Aldrich Corp., (St. Louis, MO, USA), and Nacalai Tesque Co., Ltd. (Kyoto, Japan).

### Site-directed mutagenesis

Site-directed mutagenesis (His320 being converted to Asp) was carried out on *oxdA* by means of an overlap extension PCR protocol [22, 44]. To construct OxdA(H320D), two PCRs, with plasmid pET-*oxdA* as the template, were performed with primer pairs H320D-S (5’-CGGCTGTACGACGAGGTATCGGTCTCGGAC-3’) plus T7T (5’-AGAGGGATATCACTCAGCATAAT-3’), and T7P (5’- TAATACGACTCACTATAGGGAGA-3’) plus H320D-AS (5’-GATACCTCGTCGTACAGCCGCAACTTTTTC-3’). These reactions produced 3′ and 5′ fragments of *oxdA*, respectively, whose sequences overlapped by 20 base pairs at the mutation. A second round of PCR was performed by mixing equimolar amounts of the first round products, followed by amplification between primers T7P and T7T to produce the full-length *oxdA*. The second-round product was digested with NdeI and SalI, ligated into expression vector pET-24a(+), and then sequenced. The clone with the sequence for the desired His320 → Asp mutation [pET-*oxdA(H320D)*] or pET-*oxdA(H320A)* (ref) was transformed into *Escherichia coli* BL21-CodonPlus(DE3)-RIL. The recombinant cells were used for the overproduction and purification of the mutant OxdAs (H320D and H320A).

### Expression and purification of recombinant OxdA and its mutants

Recombinant OxdA and its mutants were overexpressed and purified in the same manner as described previously [28] with some modifications. All steps were performed at 0–4°C. Potassium phosphate buffer (KPB) (pH 7.0) was used throughout the purification. Centrifugation was carried out for 30 min at 15,000 × *g*.

The cells were harvested by centrifugation, washed twice with 100 mM buffer, and then disrupted by sonication (Insonator model 201M; Kubota, Tokyo, Japan) to prepare a cell-free extract. Cell debris was removed by centrifugation. The resulting supernatant was fractionated with ammonium sulfate (30–60% saturation), followed by dialysis against 10 mM buffer. The dialyzed solution was applied to a DEAE-Sephacel column (4 × 20 cm) (GE Healthcare UK Ltd., Bucks, UK) equilibrated with 10 mM buffer. Protein was eluted from the column with 1.0 liter of 10 mM buffer, the concentration of KCl being increased linearly from 0 to 0.5 M. Each fraction was analyzed by SDS-PAGE. The fractions, which contained the band (40 kDa) corresponding to OxdA or its mutants, were collected, and then ammonium sulfate was added to give 20% saturation. The enzyme solution was placed on a TSK gel Butyl-Toyopearl 650M column (4 × 15 cm) (Tosoh Co., Tokyo, Japan) equilibrated with 10 mM buffer 20% saturated with ammonium sulfate. The enzyme was eluted by lowering the concentration of ammonium sulfate (from 20 to 0%) in 1.0 liter of the same buffer. The active fractions were combined and then precipitated with ammonium sulfate at 70% saturation. The precipitate was collected by centrifugation, dissolved in 0.1 M buffer, and then dialyzed against three changes of 5 liters of 1 mM buffer (pH 7.0). After centrifugation, the enzyme solution was loaded onto a Cellulofine HAp column (4 × 5 cm) (Seikagaku Kogyo Co., Tokyo, Japan) equilibrated with 1 mM buffer (pH 7.0). The column was eluted with a linear gradient, 1–100 mM, of the buffer (pH 7.0). The resultant solution was dialyzed against 10 mM buffer (pH 7.0) and then centrifuged. The fractions, which contained OxdA or each of its mutants, were collected and concentrated by ultrafiltration using an Amicon YM-30 membrane (Millipore Corp., Bedford, MA, USA) and a Vivaspin 30,000 molecular weight cut-off PES membrane (Sartorius K.K., Tokyo, Japan). The enzyme solution was loaded onto a Superdex 200 column (1 × 30 cm) (GE Healthcare UK Ltd.) equilibrated with 50 mM buffer including 0.15 M KCl. The active fractions were collected. The homogeneity of the purified recombinant OxdAs (WT, H320D and H320A) was confirmed by SDS-PAGE.

### Enzyme assays

#### Measurement of catalase activity

In the case of standard assay A, enzyme activity was assayed by measuring the increase in O_2_ with an oxygen electrode (Hansatech Instruments Ltd., Norfolk, UK), which monitored the O_2_ concentration [40]. The reaction mixture was composed of various concentrations (10–400 mM) of H_2_O_2_, 100 mM KPB (pH 7.0), and an enzyme [2 μM OxdA(WT), 0.5 μM OxdA(H320D) or 1 μM OxdA(H320A)] in a final volume of 1 ml. The reaction was initiated by injecting the enzyme solution into an electrode cuvette and was carried out at 30°C. One unit of catalase activity was defined as the amount of enzyme that catalyzed the production of 0.5 μmol of O_2_/min (equal to the consumption of 1 μmol of H_2_O_2_/min) under the standard assay A conditions. Specific activity is expressed as units/mg protein. *k*_cat_ values were calculated using *V*_max_ values and a *M*_r_ of 40,127 for OxdA. This assay was used to routinely measure catalase activity, unless otherwise noted.

#### Measurement of peroxidase activity

The standard assay B mixture comprised 100 mM KPB (pH 7.0), an excess (10–400 mM) of H_2_O_2_ for enzymes, various concentrations (0.01–10 mM) of guaiacol, and an enzyme [1 μM OxdA(WT), 0.25 μM OxdA(H320D) or 1 μM OxdA(H320A)] in a total volume of 100 μl. The reaction was started by the addition of the enzyme and was carried out for 1 min at 30°C. The production of tetra-guaiacol, which is the reaction product, was determined from the increase in the absorbance at 470 nm [(ε) 3.8 × 10^3^ M^-1^ cm^-1^] [45]. One unit of peroxidase activity was defined as the amount of enzyme that catalyzed the production of 0.25 μmol of tetra-guaiacol/min (equal to the consumption of 1 μmol of H_2_O_2_/min) under the standard assay B conditions.

The standard assay C mixture comprised 100 mM KPB (pH 7.0), an excess (1–100 mM) of H_2_O_2_ for enzymes, various concentrations (0.01–1 mM) of 2,2'-azino-bis (3-ethylbenzothiazoline-6-sulfonic acid ammonium salt) (ABTS), and an enzyme [1 μM OxdA(WT), 0.25 μM OxdA(H320D) or 1 μM OxdA(H320A)] in a total volume of 100 μl. The reaction was started by the addition of the enzyme and was carried out for 1 min at 30°C. The production of an ABTS radical, which is a reaction product, was determined from the increase in the absorbance at 430 nm [(ε) 1.4 × 10^4^ M^-1^ cm^-1^] [45]. One unit of peroxidase activity was defined as the amount of enzyme that catalyzed the production of 1 μmol of ABTS radical/min (equal to the consumption of 1 μmol of H_2_O_2_/min) under the standard assay C conditions. Specific activity is expressed as units/mg protein. *k*_cat_ values were calculated using *V*_max_ values and a *M*_r_ of 40,127 for OxdA.

#### Measurement of peroxygenase activity

The standard assay D mixture comprised 15% (v/v) ethanol, 100 mM KPB (pH 7.0), an excess (1–100 mM) of H_2_O_2_ for enzymes, various concentrations (0.01–0.5 mM) of 1-methoxynaphthalene (1-MN), and an enzyme [1 μM OxdA(WT) 0.25 μM OxdA(H320D), or 1 μM OxdA(H320A)] in a total volume of 100 μl. The reaction was started by the addition of the enzyme and was carried out for 1 min at 30°C. The production of Russig’s blue, which is a reaction product, was determined from the increase in the absorbance at 610 nm [(ε) 1.45 × 10^4^ M^-1^ cm^-1^] [46, 47]. One unit of peroxygenase activity was defined as the amount of enzyme that catalyzed the consumption of 0.25 μmol of Russig’s blue/min (equal to the consumption of 1 μmol of H_2_O_2_/min) under the standard assay D conditions. As for OxdA(H320D), the production of 1-methoxy-2-naphthalenol (described latter), which is a reaction product, was determined from the increase in the absorbance at 334 nm [(ε) 4.76 × 10^3^ M^-1^ cm^-1^], which was determined for purified 1-methoxy-2-naphthalenol in this study. One unit of peroxygenase activity was defined as the amount of enzyme that catalyzed the consumption of 1 μmol of 1-methoxy-2-naphthalenol/min (equal to the consumption of 1 μmol of H_2_O_2_/min) under the standard assay D conditions. Specific activity is expressed as units/mg protein. *k*_cat_ values were calculated using *V*_max_ values and a *M*_r_ of 40,127 for OxdA.

#### Spectral measurements

The UV-Vis spectroscopy measurements were carried out using a UV-1700 (Shimadzu Co., Kyoto, Japan). The reaction mixture was composed of 100 mM KPB (pH 7.0), 1 mM H_2_O_2_, 0.5 mM 1-MN and 0.5 μM enzyme. For measurements, the reaction was started by the addition of the enzyme, and the mixed reaction solutions were introduced into the optical cell, and absorption measurements were started at 28°C.

### HPLC and LC/MS analyses

A sample was analyzed by HPLC with a Shimadzu Prominence system including a photodiode array detector (SPD-M20A), and an LCMS-8030 (Shimadzu Co.) equipped with a Kinetex 1.7 μm C18 100Å column (2.1 × 50 mm; Phenomenex Co., Ltd., LA, USA). The HPLC conditions were as follows: flow rate, 0.4 ml/min; solvent A, 0.1% (v/v) HCOOH; and solvent B, acetonitrile. After column equilibration with 10% solvent B, a linear gradient system of solvent B, 10% to 100%, was applied over 5 min, followed by 100% solvent B for 3 min.

### Purification and NMR analysis of the peroxygenase reaction product with OxdA(H320D)

After the peroxygenase reaction with OxdA(H320D) at 25°C for 30 min, the reaction mixture was extracted with ethyl acetate and concentrated by evaporation. The reaction product dissolved in water was further purified by HPLC [TSK-gel ODS-80Ts (7.8 by 300 mm; Tosoh Co.), with a linear gradient of 10~100% (v/v) acetonitrile in water]. The peak fractions were collected and concentrated to dryness (0.742 mg). Nuclear magnetic resonance (NMR) spectra of the product dissolved in CDCl_3_ were measured with an AVANCE-600 NMR spectrometer (Bruker, Ettlingen, Germany), and calibrated with CDCl_3_.

### Measurement of reduction potentials of OxdAs (WT, H320A and H320D)

The reduction potentials of OxdAs (WT, H320A an H320D) were evaluated by cyclic voltammetry. A gold electrode (polycrystalline Au, 3 mm in diameter; ALS Co., Ltd, Tokyo, Japan) was used as a working electrode. The surface of the gold electrode was polished with an alumina slurry (0.3 μm), sonicated and rinsed with distilled water. Cyclic voltammetry was carried out on an electrochemical analyzer (CHI 611A, ALS Co., Ltd.). The measurements were carried out in 100 mM KPB (pH 7.0) in the presence and absence of an enzyme (10 μM) at a scan rate of 5 mV/s and room temperature (25 ± 1°C). Prior to each experiment, the solutions were bubbled with argon gas for 10 min. A platinum wire counter electrode and an Ag|AgCl|KCl (sat.) reference electrode were used.

### Analytical methods

SDS-PAGE was performed in a 12% polyacrylamide slab gel according to Laemmli [48]. The gel was stained with Coomassie brilliant blue R-250. The molecular mass of the subunit of the mutant enzyme was determined from the relative mobilities of marker proteins, phosphorylase *b* (94 kDa), bovine serum albumin (67 kDa), ovalbumin (43 kDa), carbonic anhydrase (30 kDa), soybean trypsin inhibitor (20.1 kDa), and α-lactalbumin (14.4 kDa).

## Results

### Detection of catalase activity of OxdAs (WT, H320D and H320A)

**Fig 1:**
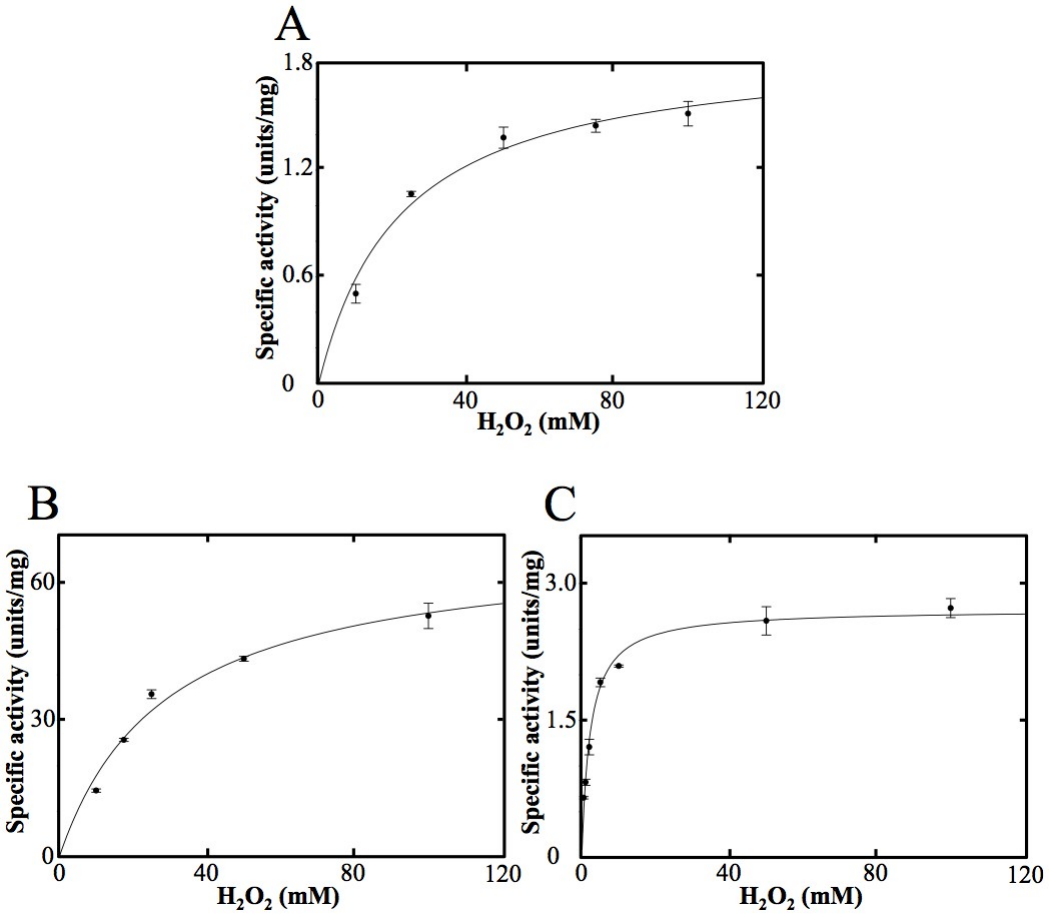
Michaelis—Menten kinetics of the catalase activity of OxdAs. The reactions were carried out under the “standard assay A” conditions as described under “Materials and Methods.” For all data points, values are means ± mean error. OxdA(WT) (A), OxdA(H320D) (B) and OxdA(H320A) (C).

OxdA catalyzes the dehydration of aldoximes to the corresponding nitriles, but not redox reactions. First, we examined the catalase activity of OxdA by observing the increase in O_2_ by using an oxygen electrode. In the reaction mixture containing 10 mM H_2_O_2_ and 2 μM OxdA, the production of O_2_ (S1A Fig) was detected. On the other hand, an increase in O_2_ was not detected in the reaction mixture containing heat-treated OxdA (for 10 min at 98°C), which did not give the specific absorption peak of heme (S2A Fig). These findings for the first time indicated that OxdA catalyzes a catalase reaction. Then, we determined the Michaelis—Menten kinetics of the activity at the following concentrations of H_2_O_2_ (10–100 mM) (Fig 1A). The apparent *K*_m_ and *V*_max_ values of OxdA were found by means of Hanes-Woolf plots (S3A Fig) to be 22 ± 4 mM and 1.9 ± 0.1 units/mg, respectively (Table 1).

**Table 1:** Catalase activity of OxdAs (WT, H320D and H320A).

Enzyme	*K*_m_ (mM)	*V*_max_ (units/mg)	*k*_cat_ (s^-1^)	*k*_cat_/ *K*_m_ (s^-1^ mM^-1^)
OxdA(WT)	22 ± 4	1.9 ± 0.1	1.3 ± 0.1	0.057
OxdA(H320D)	29 ± 4	69 ± 4	46 ± 3	1.6
OxdA(H320A)	2.3 ± 0.2	2.7 ± 0.1	1.8 ± 0.0	0.80

The reaction was carried out using “standard assay A” described under “Materials and Methods.” Apparent *K*_m_ values of OxdAs (WT, H320D and H320A) for H_2_O_2_, and their apparent *V*_max_ values were obtained from Hanes-Woolf plots. *k*_cat_ values were calculated using *V*_max_ values and a *M*_r_ of 40,127 for OxdA.

We constructed a mutant OxdA (His320 being converted to Asp), OxdA(H320D), to mimic the chloroperoxidase (CPO) from fungus *Caldariomyces fumago* [41, 43]. In the chloroperoxidase reaction and the catalase reaction [41], the Asp residue located in the distal site of heme of the His64 → Asp myoglobin mutant mimicking CPO [39, 40] has been suggested to enhance the formation of a ferryl porphyrin cation radical (compound I) as a reaction intermediate in the catalase reaction. In addition, to examine the effect of removal of the charge of the 320th residue, we used the other mutant OxdA (His320 being converted to Ala), OxdA(H320A) (29).

We measured the catalase activity of mutant OxdAs (H320D and H320A), and found that both mutant enzymes (H320D and H320A) also catalyzed a catalase reaction (S1B and S1C Fig), whereas heat-treated ones (for 10 min at 98°C) not exhibiting the specific absorption peak of heme (S2B and S2C Fig) catalyzed no catalase reaction. Their kinetic parameters were determined by means of Hanes-Woolf plots (S3B and S3C Fig) at the following concentrations of H_2_O_2_, 10–100 mM (Fig 1B and 1C). The kinetic parameters of OxdA(H320D) were found to be *K*_m_ value 29 ± 4 mM and *V*_max_ value 69 ± 4 units/mg, and those of OxdA(H320A) to be *K*_m_ value 2.3 ± 0.2 mM and *V*_max_ value 2.7 ± 0.1 units/mg (Table 1). These findings demonstrate that the *V*_max_ value (69 ± 4 units/mg) of OxdA(H320D) was about 30 times higher than those of the wild-type OxdA [namely, OxdA(WT)] (units/mg) and OxdA(H320A) (1.9 ± 0.1 and 2.7 ± 0.1 units/mg), respectively, while the *K*_m_ value (2.3 ± 0.2 mM) of OxdA(H320A) for H_2_O_2_ was about 10 times lower than those of OxdA(WT) and OxdA(H320D) (22 ± 4 and 29 ± 4 mM), respectively.

### Detection of peroxidase activity of OxdAs (WT, H320D and H320A)

Next, we examined the peroxidase activity (SH_2_ + H_2_O_2_ → S + 2H_2_O) of the OxdAs (WT, H320D and H320A), which is one of the redox activities. For the peroxidase assay, two different substrates were used.

**Fig 2:**
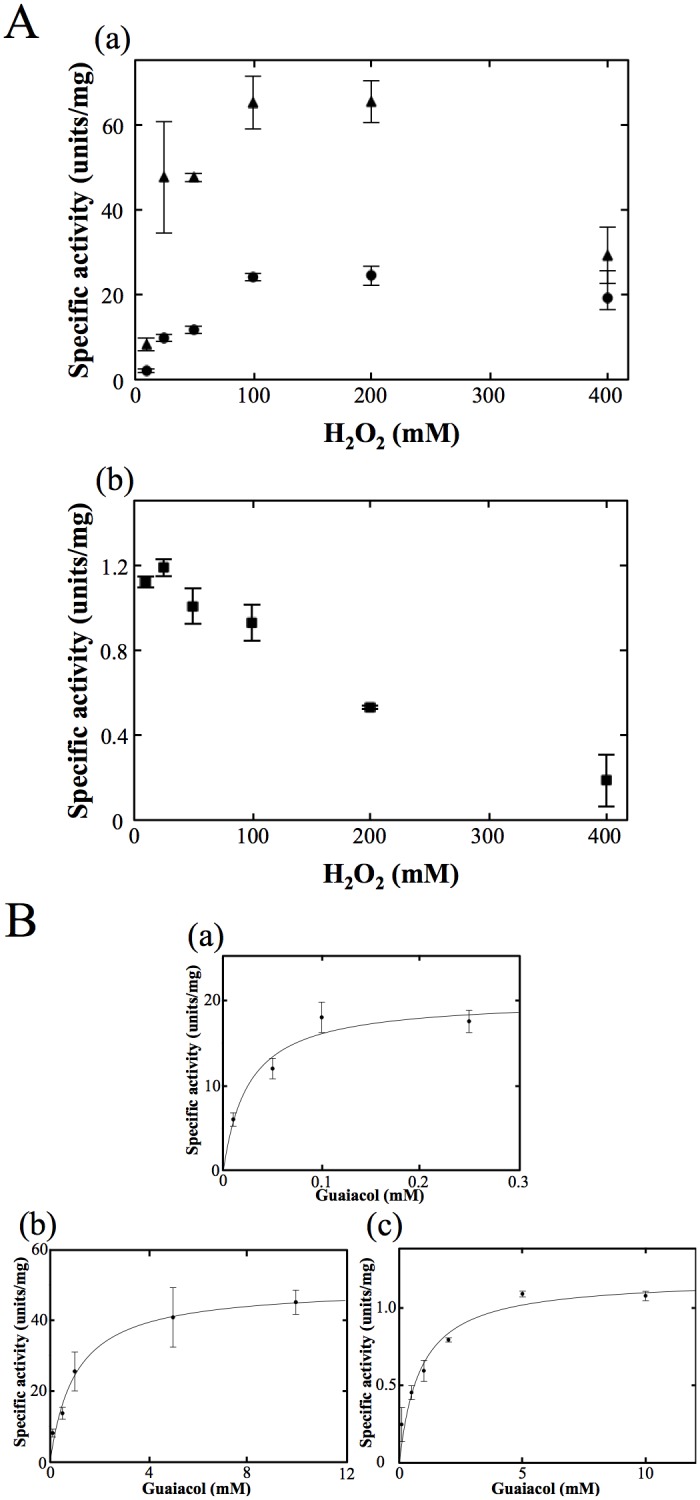
Analyses of peroxidase activities of OxdAs in the presence of guaiacol. (A) The peroxidase activities of OxdAs depending on the H_2_O_2_ concentration. OxdA(WT) [(a) black circles], OxdA(H320D) [(a) black triangles], and OxdA(H320A) [(b) black square]. (B) Michaelis—Menten kinetics of the peroxidase activity of OxdAs. OxdA(WT) (a), OxdA(H320D) (b) and OxdA(H320A) (c). The reactions with guaiacol as a substrate were carried out under the “standard assay B” conditions as described under “Materials and Methods.” For all data points, values are means ± mean error.

(i) We measured the peroxidase activity of OxdA(WT) using guaiacol as a substrate. In the reaction mixture containing 25 mM H_2_O_2_, 10 mM guaiacol and 1 μM OxdA under the standard assay B conditions, an increase in absorbance at 470 nm, which is the absorption maximum for a reaction product (tetra-guaiacol) [45], was observed (S4A Fig). On the other hand, this increase was not observed on the addition of heat-treated OxdA (for 10 min at 98°C). These findings, for the first time, indicated that OxdA catalyzes a peroxidase reaction. In the case of OxdA, the peroxidase activity would be strongly influenced by its catalase activity. Furthermore, a large amount of H_2_O_2_ present in the reaction mixture would generally inhibit various enzymatic activities due to its strong oxidizing power [49]. Thus, we measured each peroxidase activity in a reaction mixture containing various concentrations of H_2_O_2_ (10~400 mM) and excess guaiacol (10 mM) [Fig 2A(a)]. The peroxidase activity was inhibited by H_2_O_2_ in excess of 200 mM [Fig 2A(a)], indicating that 100 mM H_2_O_2_ is suitable for measurement of the peroxidase activity. The tendency of H_2_O_2_ concentration-dependent inhibition of the peroxidase activity was similar to that of the catalase activity (S5 Fig). These inhibitions would be caused by the inactivation of OxdA due to the strong oxidizing power of H_2_O_2_. Therefore, we determined the Michaelis—Menten kinetics of the peroxidase activity in a reaction mixture containing 100 mM H_2_O_2_ [Fig 2B(a)]. The apparent *K*_m_ value for guaiacol and the *V*_max_ value of OxdA were found by means of Hanes-Woolf plots (S6A Fig) to be 0.026 ± 0.010 mM and 20 ± 2 units/mg, respectively (Table 2). Moreover, we found that a peroxidase reaction was catalyzed by the mutant OxdAs (H320D and H320A) (S4 and S4C Fig), but not by the heat-treated ones (for 10 min at 98°C). We determined the suitable concentrations of H_2_O_2_ of mutant OxdAs (H320D and H320A) to be 100 and 25 mM for the peroxidase assay (Fig 2A), respectively. The kinetic parameters of mutant OxdAs (H320D and H320A) were determined by means of Hanes-Woolf plots (S6 and S6C Fig) to be *K*_m_ values for guaiacol 1.0 ± 0.4 and 0.84 ± 0.17 mM, and *V*_max_ values 50 ± 5 and 1.2 ± 0.1 units/mg [Fig 2B(b) and 2B(c)] (Table 2), respectively. These findings demonstrated that the *V*_max_ value (50 ± 5 units/mg) of OxdA(H320D) was about 2.5 times higher than that (20 ± 2 units/mg) of OxdA(WT), however, the *V*_max_ value (1.2 ± 0.1 units/mg) of OxdA(H320A) was about 20 times lower than that (20 ± 2 units/mg) of OxdA(WT). On the other hand, the *K*_m_ values (1.0 ± 0.4 and 0.84 ± 0.17 mM) of mutant OxdAs (H320D and H320A) for guaiacol were dozens of times lower than that (0.026 ± 0.010 mM) of OxdA(WT).

**Table 2:** Peroxidase activity of OxdAs using guaiacol as a substrate.

Enzyme	*K*_m_ (mM)	*V*_max_ (units/mg)	*k*_cat_ (s^-1^)	*k*_cat_/ *K*_m_ (s^-1^ mM^-1^)
OxdA(WT)	0.026 ± 0.010	20 ± 2	14 ± 1	530
OxdA(H320D)	1.0 ± 0.4	50 ± 5	33 ± 3	32
OxdA(H320A)	0.84 ± 0.17	1.2 ± 0.1	0.81 ± 0.05	0.97

**Fig 3:**
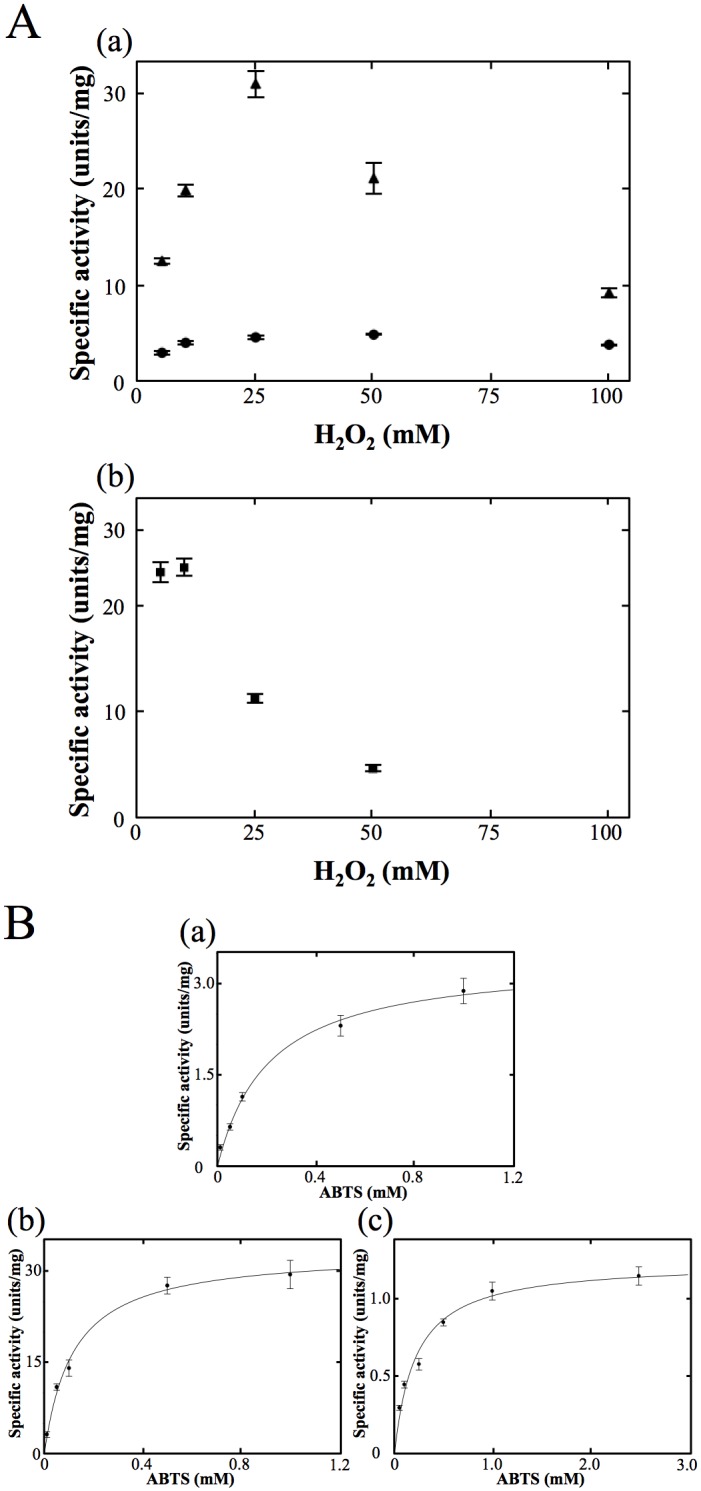
Analyses of peroxidase activities of OxdAs in the presence of ABTS. (A) The peroxidase activities of OxdAs depending on the H_2_O_2_ concentration. OxdA(WT) [(a) black circles], OxdA(H320D) [(a) black triangles], and OxdA(H320A) [(b) black square]. (B) Michaelis—Menten kinetics of the peroxidase activity of OxdAs. OxdA(WT) (a), OxdA(H320D) (b) and OxdA(H320A) (c). The reactions with ABTS as a substrate were carried out under the “standard assay C” conditions as described under “Materials and Methods.” For all data points, values are means ± mean error.

The reaction was carried out using “standard assay B” described under “Materials and Methods.” Apparent *K*_m_ values of OxdAs (WT, H320D and H320A) for guaiacol and their apparent *V*_max_ values were obtained from Hanes-Woolf plots. *k*_cat_ values were calculated using *V*_max_ values and a *M*_r_ of 40,127 for OxdA.

(ii) Instead of guaiacol, 2,2'-azino-bis (3-ethylbenzothiazoline-6-sulfonic acid ammonium salt) (ABTS) was used as a substrate. With the reaction mixture consisting of 25 mM H_2_O_2_, 2.5 mM ABTS and 1 μM OxdA(WT) under the standard assay C conditions (S7 Fig), we observed an increase in the absorbance at 430 nm due to the absorption maximum for a peroxidase reaction product (ABTS radical) [45]. As for the peroxidase assay with guaiacol as a substrate, the peroxidase activity in the reaction mixture containing various concentrations of H_2_O_2_ (1~100 mM) and excess ABTS (2.5 mM) for OxdA was measured [Fig 3A(a)]. Even with 100 mM H_2_O_2_, surprisingly, the peroxidase activity using ABTS as a substrate was inhibited, indicating the suitable concentration of H_2_O_2_ was 25 mM for the assay. The tendency of H_2_O_2_ concentration-dependent inhibition was different from those for the catalase (S5 Fig) and peroxidase (using guaiacol as a substrate) (Fig 2A) reactions. Inhibition of the peroxidase activity (using ABTS as a substrate) by a low concentration of H_2_O_2_ would be caused by competition between the catalase and peroxidase activities (for further details, please refer to ''Discussion''). On determination of the Michaelis—Menten kinetics of the activity in the reaction mixture containing 25 mM H_2_O_2_ [Fig 3B(a)], the apparent *K*_m_ for ABTS and *V*_max_ values of OxdA(WT) were found by means of Hanes-Woolf plots (S8A Fig) to be 0.21 ± 0.04 mM and 3.4 ± 0.2 units/mg, respectively (Table 3). Furthermore, we succeeded in the detection of peroxidase activity of mutant OxdAs (H320D and H320A) (S7 Fig). The suitable concentrations of H_2_O_2_ for OxdAs (H320D and H320A) were determined to be 25 and 10 mM for the peroxidase assay (Fig 3A), respectively, and the kinetic parameters were determined by means of Hanes-Woolf plots (S8 and S8C Fig) to be *K*_m_ values 0.12 ± 0.02 and 0.21 ± 0.03 mM for ABTS, and *V*_max_ values 33 ± 2 and 1.3 ± 0.1 units/mg, respectively [Fig 3B(b) and 3Bc)] (Table 3). These findings demonstrated that the *V*_max_ value (33 ± 2 units/mg) of OxdA(H320D) was about 10 times higher than that (3.4 ± 0.2 units/mg) of OxdA(WT), while that of OxdA(H320A) (1.3 ± 0.1 units/mg) was about 3 times lower than that of OxdAs (WT). On the other hand, the *K*_m_ value (0.12 ± 0.02 mM) of OxdA(H320D) for ABTS was nearly half of those (0.21 ± 0.04 and 0.21 ± 0.03 mM) of OxdAs (WT and H320A).

**Table 3:** Peroxidase activity of OxdAs using ABTS as a substrate.

Enzyme	*K*_m_ (mM)	*V*_max_ (units/mg)	*k*_cat_ (s^-1^)	*k*_cat_/ *K*_m_ (s^-1^ mM^-1^)
OxdA(WT)	0.21 ± 0.04	3.4 ± 0.2	2.3 ± 0.1	11
OxdA(H320D)	0.12 ± 0.02	33 ± 2	22 ± 1	190
OxdA(H320A)	0.21 ± 0.03	1.3 ± 0.1	0.85 ± 0.07	4.0

The reaction was carried out using “standard assay C” described under “Materials and Methods.” Apparent *K*_m_ values of OxdAs (WT, H320D and H320A) for ABTS and their apparent *V*_max_ values were obtained from Hanes-Woolf plots. *k*_cat_ values were calculated using *V*_max_ values and a *M*_r_ of 40,127 for OxdA.

### Detection of peroxygenase activity of OxdA(WT)

**Fig 4:**
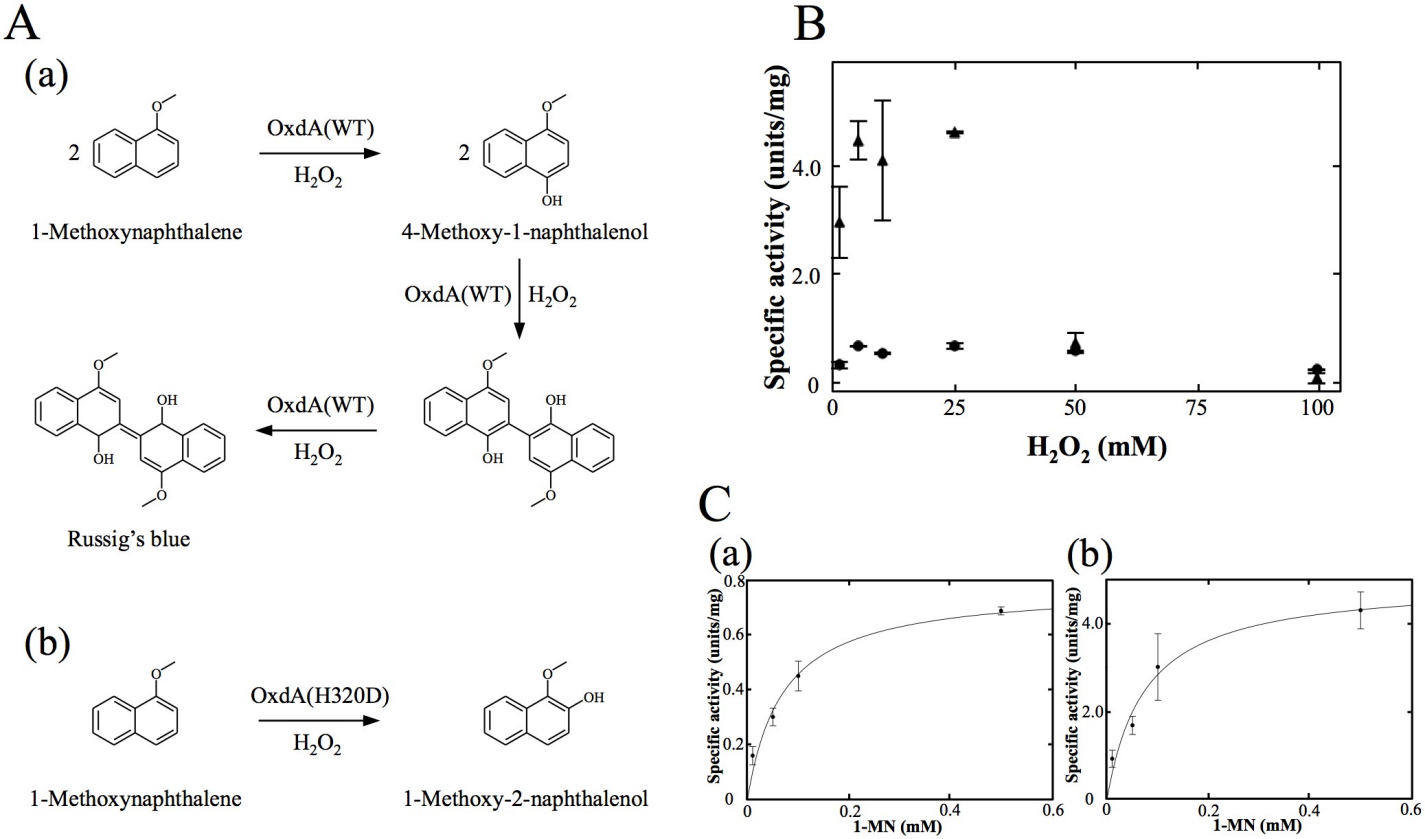
The reaction pathways and analyses of peroxygenase activities of OxdAs using 1-MN as a substrate. (A) Reaction pathway for conversion of 1-MN to Russig’s blue (a), and 1-methoxy 2-naphthalenol (b). (B) The peroxygenase activities of OxdAs depending on the H_2_O_2_ concentration. OxdA(WT) (black circles) and OxdA(H320D) (black triangles). (C) Michaelis—Menten kinetics of the peroxygenase activity of OxdAs. OxdA(WT) (a) and OxdA(H320D) (b). The reactions were carried out under the “standard assay D” conditions as described under “Materials and Methods.” For all data points, values are means ± mean error.

Next, we examined peroxygenase activity (S + H_2_O_2_ → SO + H_2_O) of OxdAs (WT, H320D and H320A) in order to discover a third redox activity. To measure peroxygenase activity, we used 1-methoxynaphthalene (1-MN) as a substrate, and measured the absorbance at 610 nm due to the absorption maximum of the reaction product (Russig’s blue) [46, 47] [Fig 4A(a)]. In the reaction mixture containing 5 mM H_2_O_2_, 0.5 mM 1-MN and 1 μM OxdA, the marked production of Russig’s blue was observed under the standard assay D conditions (S9 Fig). With the use of heat-treated OxdA (for 10 min at 98°C), however, this increase in absorbance at 610 nm was not observed. These findings for the first time indicated that OxdA catalyzes a peroxygenase reaction. As for the peroxidase assays, we determined the suitable amount of H_2_O_2_ to be 5 mM for the assay (Fig 4B), by measuring each peroxygenase activity in the reaction mixture containing various concentrations of H_2_O_2_ (1–100 mM) and 0.5 mM 1-MN, this concentration giving near saturation. The suitable H_2_O_2_ concentration was different from those of the catalase and peroxidase activities (for further details, please refer to ''Discussion''). The Michaelis—Menten kinetics of the activity were determined with a suitable concentration of H_2_O_2_ [Fig 4C(a)]. The apparent *K*_m_ for 1-MN and *V*_*max*_ values of OxdA(WT) were found by means of Hanes-Woolf plots (S10A Fig) to be 0.070 ± 0.017 mM and 0.77 ± 0.06 units/mg, respectively (Table 4).

**Table 4:** Peroxygenase activity of OxdAs (WT and H320D).

Enzyme	*K*_m_ (mM)	*V*_max_ (units/mg)	*k*_cat_ (s^-1^)	*k*_cat_/ *K*_m_ (s^-1^ mM^-1^)
OxdA(WT)	0.070 ± 0.017	0.77 ± 0.06	0.52 ± 0.04	7.4
OxdA(H320D)	0.075 ± 0.030	5.0 ± 0.7	3.3 ± 0.4	44

The reaction was carried out using “standard assay D” described under “Materials and Methods.” Apparent *K*_m_ values of OxdAs (WT and H320D) for 1-MN and their apparent *V*_max_ values were obtained from Hanes-Woolf plots. *k*_cat_ values were calculated using *V*_max_ values and a *M*_r_ of 40,127 for OxdA

### Unexpected peroxygenase reaction of OxdAs (H320D and H320A)

**Fig 5:**
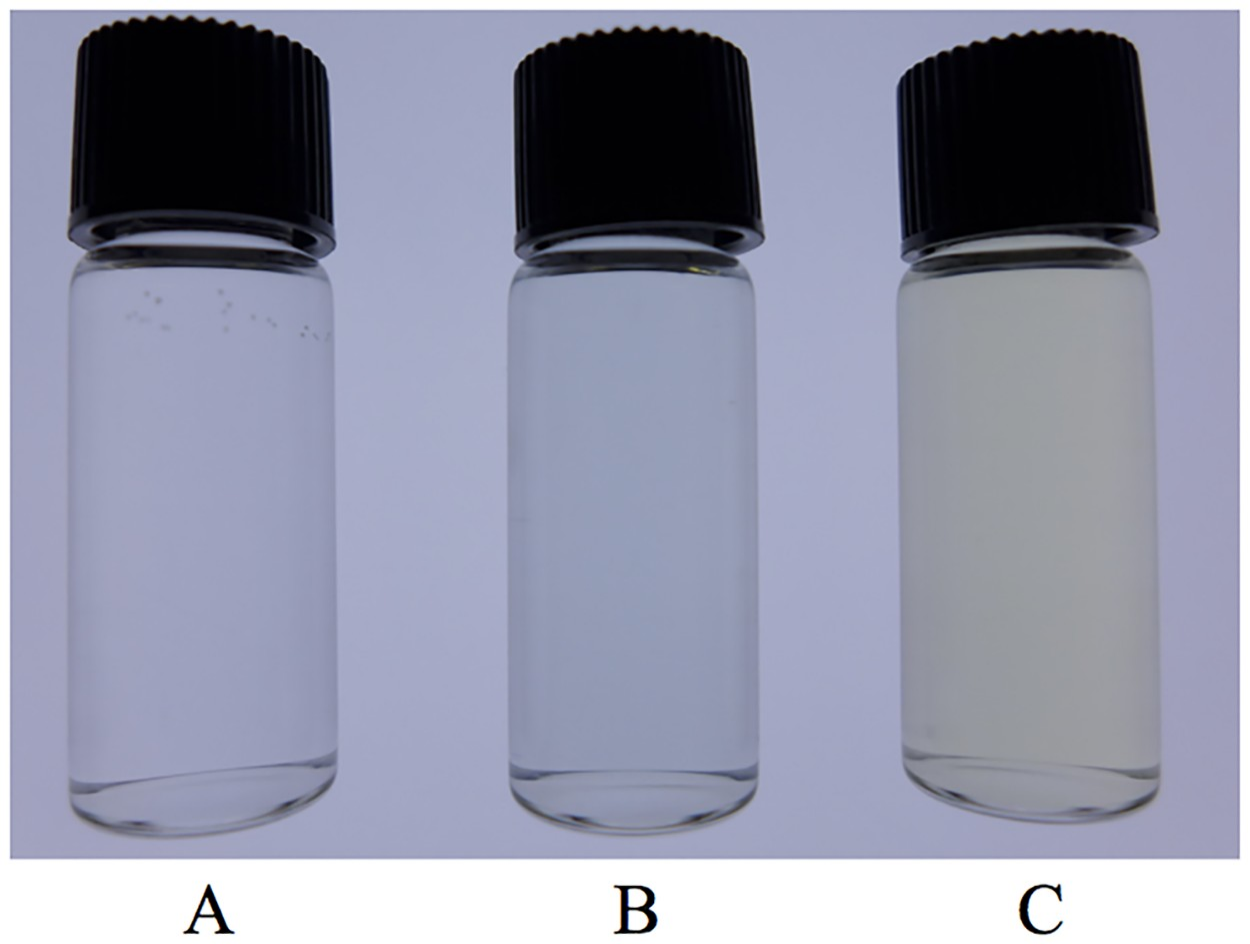
Photograph of the peroxygenase reaction mixtures containing OxdA(WT) and OxdA(H320D). The reaction mixture containing 0.5 mM 1-MN and 5 mM H_2_O_2_ in the absence of an enzyme (A), 10 μM OxdA(WT) (B), or OxdA(H320D) (C) at pH 7.0 and 28°C after mixing at 15 min.

**Fig 6:**
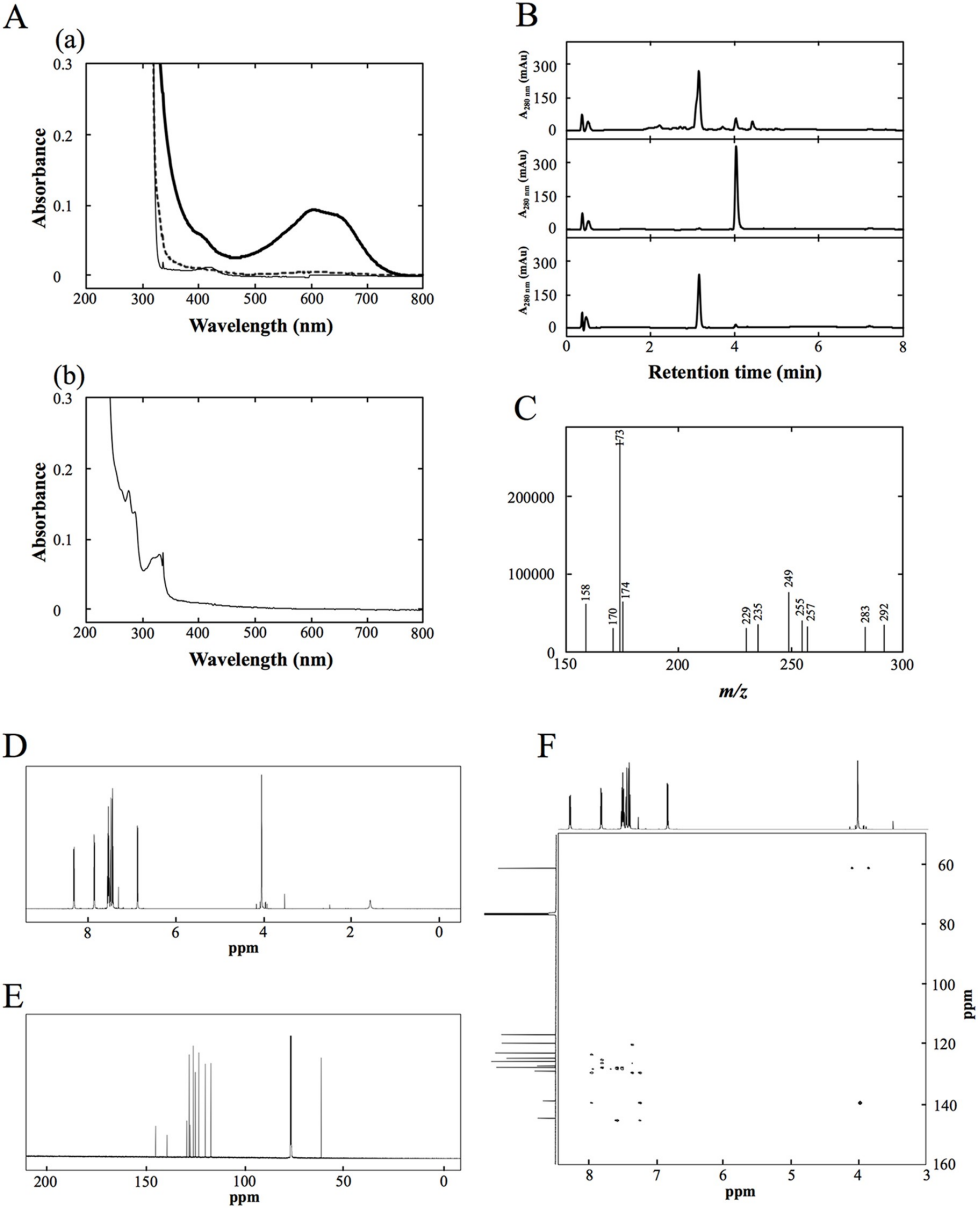
Absorption spectroscopy, HPLC, LC/MS and NMR analyses of the purified product. (A) (a)Absorption spectra of reaction mixture containing 0.5 mM 1-MN, 5 mM H_2_O_2_ and 1 μM OxdA(WT) (thick line), OxdA(H320D) (broken line), or OxdA(H320A) (thin line) at pH 7.0 and 28°C after mixing at 10 min. (b) Absorption spectrum of the purified 1-methoxy-2-naphthalenol in an ethanol solution (0.05 mM). (B) HPLC elution profiles of a chloroform extract of the reaction mixture containing 1-MN and OxdA(H320D) (Top), a chloroform extract of the reaction mixture containing 1-MN and heat-treated OxdA(H320D) (Middle), and the purified reaction product (Bottom). (C) The mass spectrum on LC-ESI-MS in the negative ion mode of the peak of the purified new product. The major mass peak at *m/z* 173 corresponds to [M—H]^-^ of 1-methoxy-2-naphthalenol. (D) ^1^H NMR spectrum of purified product. (E) ^13^C NMR spectrum of the purified product. (F) HMBC spectrum of the purified product. The experimental procedures were carried out by the methods described under “Materials and Methods.”

For OxdA(H320D), we also measured its peroxygenase activity under the standard assay D conditions. In the reaction mixture containing OxdA(H320D), we found that Russig’s blue was not produced, but that a different compound was, based on the characteristic color of the reaction mixture [Figs 5 and 6A(a)]. Therefore, we purified the new reaction product by HPLC (Fig 6B), and measured its mass spectrum by LC/MS as described under “Materials and Methods.” The major mass peak derived from the purified product was found at *m/z* 173 in the negative ion mode (Fig 6C). These findings demonstrated that the molecular mass of the new reaction product is 174. Furthermore, NMR spectra of the purified product were measured [^1^H-NMR (600 MHz, CDCl_3_): *δ*8.30 (m, 1H, H-8), 7.84 (m, 1H, H-5), 7.52 (m, 1H, H-6 and 7), 7.48 (d, 1H, J = 11.8 Hz, H-4), 7.44 (t, 1H, J = 22.1 Hz, H-3), 6.85 (d, 1H, J = 7.44 Hz, H-2), 4.00 (s, 3H, O-CH_3_); ^13^C-NMR (150 MHz, CDCl_3_): *δ* 145.0 (C-2), 139.0 (C-1), 129.6 (C-4a), 128.3 (C-5), 127.8 (C-8a), 126.3 (C-7), 125.3 (C-4), 123.5 (C-6), 120.3 (C-8), 117.4 (C-3), 61.7 (O-CH_3_)] (Fig 6D–6F). Based on these findings, the new product of the peroxygenase reaction with OxdA(H320D) was identified as 1-methoxy-2-naphthalenol.

Next, we measured the absorption spectrum of the purified product (0.05 mM) [Fig 6A(b)], and then determined the molar extinction coefficients of 1-methoxy-2-naphthalenol at 280 nm (9.38 × 10^3^ M^-1^cm^-1^) and 334 nm (4.76 × 10^3^ M^-1^cm^-1^). By use of the latter coefficient value, we measured the peroxygenase activity of OxdA(H320D) using the increase in the absorbance at 334 nm, which does not overlap the absorption at 280 nm for various proteins including OxdA, under the standard assay D conditions. As for the peroxidase assays, we observed each peroxygenase activity in the reaction mixture containing various concentrations of H_2_O_2_ (1~100 mM) and 0.5 mM 1-MN, this concentration giving near saturation, and revealed that the suitable concentration of H_2_O_2_ was 5 mM for the assay (Fig 4B). The determined suitable H_2_O_2_ concentration was different from those of the catalase and peroxidase activities (for further details, please refer to ''Discussion''). With the suitable concentration of H_2_O_2_, we determined the kinetic parameters of OxdA(H320D) by means of Hanes-Woolf plots (S10B Fig) to be *K*_m_ value 0.075 ± 0.030 mM for 1-MN and *V*_max_ value 5.0 ± 0.7 units/mg [Fig 4C(b)] (Table 4). These findings demonstrated that the *V*_max_ value (5.0 ± 0.7 units/mg) of OxdA(H320D) was about 6 times higher than that (0.77 ± 0.06 units/mg) of OxdA(WT), while the *K*_m_ value (0.075 ± 0.030 mM) of OxdA(H320D) for 1-MN was similar to that (0.070 ± 0.017 mM) of OxdA(WT).

Finally, we measured the peroxygenase activity of OxdA(H320A) by observing the increase in absorbance at 610 nm (Russig’s blue) and 334 nm (1-methoxy-2-naphthalenol) in the reaction mixture containing 5 mM H_2_O_2_ and 0.5 mM 1-MN and 1 μM OxdA(H320A), but both values did not increase (S9 and S11 Figs). Even if a 10-fold higher amount (10 μM) of OxdA(H320A) was added, no increase in either value was observed. Furthermore, we carried out the absorption spectrum analysis of the peroxygenase reaction mixture of OxdA(H320A) [Fig 6A(a)]. However, no specific peak derived from another product was observed. These findings suggested that OxdA(H320A) did not catalyze the peroxygenase reaction using 1-MN as a substrate under the conditions tested.

### Evaluation of reduction potential of OxdAs

**Fig 7:**
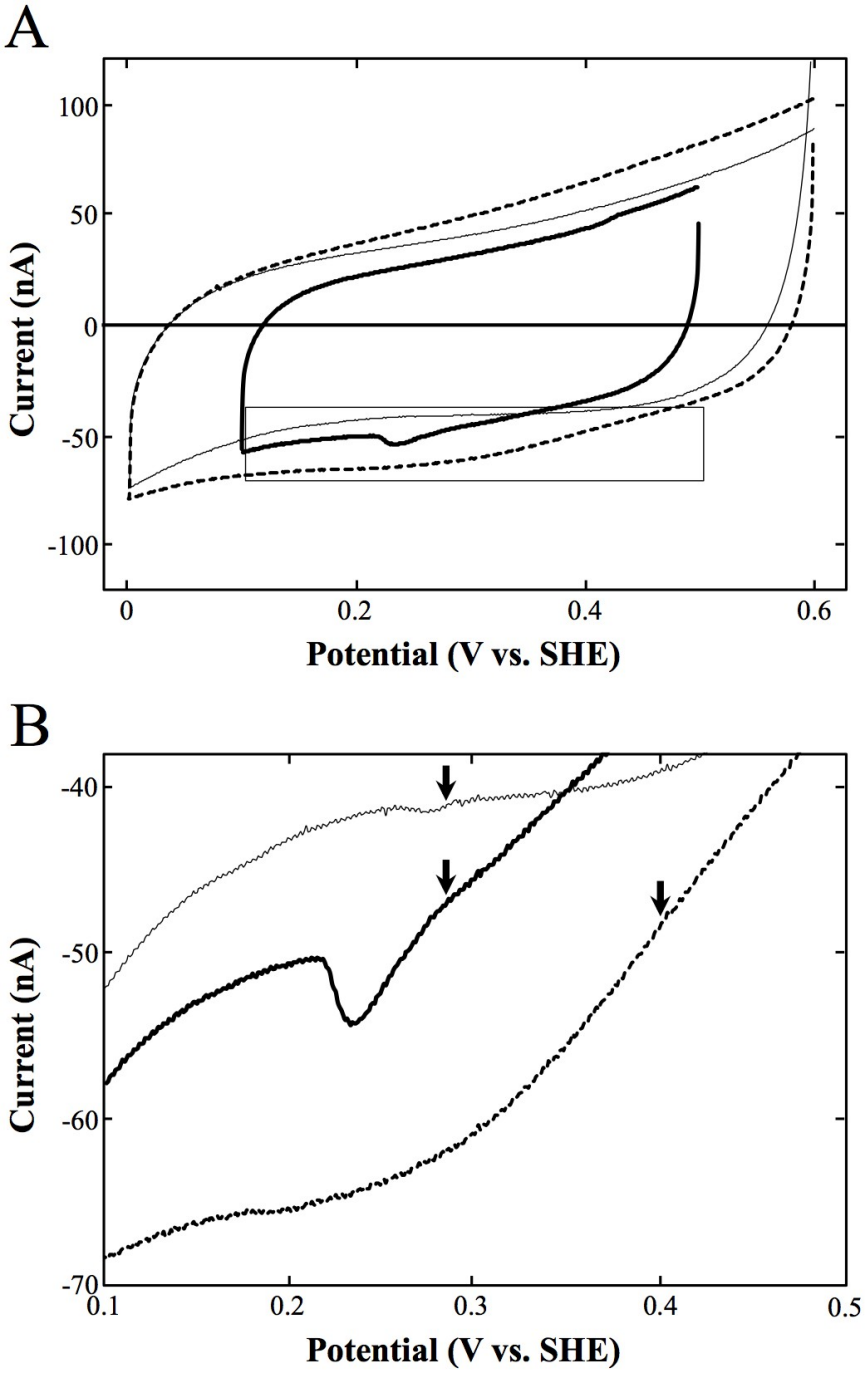
The reduction potentials of OxdAs (WT, H320D and H320A) determined by cyclic voltammetry. Electro-reduction of 10 μM OxdA(WT) (thick line), OxdA(H320D) (dotted line), or OxdA(H320A) (thin line) at a 3 mm diameter Au electrode, a scan rate of 5 mV/s and room temperature (25 ± 1°C). Overall measurements (A), zoom in its square (B), the onset potentials of OxdAs (WT, H320D and H320A) (arrows).

The reduction potentials of OxdA(WT) and its mutants were evaluated by cyclic voltammetry, which has often been used to determine the formal potential of several redox proteins. A reduction current was observed on each enzyme, as shown in Fig 7. Such peaks were not observed in the absence of the enzyme, so the reduction currents must be derived from a redox site in OxdA (most probably the protoheme site). The onset potentials, where the reduction wave begins to increase, of OxdAs (WT, H320D, and H320A) were 0.28, 0.40, and 0.28 V vs SHE, respectively.

## Discussion

### Discovery of redox activities of OxdA

Among heme enzymes, OxdA performs the uncommon catalysis of a dehydration reaction. In general, heme enzymes, which commonly catalyze redox reactions, and hemoproteins bind to an exogenous small gaseous molecule (e.g., O_2_, CO_2_ or H_2_O_2_) on the heme. On the contrary, OxdA directly binds to and activates an organic substrate on the heme in the dehydration reaction [28]. In our previous studies, we clarified various characteristics of OxdA [28–31] and elucidated the overall catalytic mechanism [32]. The present proposed reaction mechanism of OxdA for the synthesis of a nitrile is as follows: (*i*) the Ser219 residue would fix the substrate (aldoxime) and increase the basicity of the hydroxyl group of the substrate; (*ii*) the His320 residue acts as an acid-base catalyst; (*iii*) the Arg178 residue would act a proton donor-acceptor of the imidazole ring of His320; and (*iv*) the heme iron acts as an electron donor-acceptor (S12A Fig) [30, 32, 50]. However, redox catalysis of OxdA has never been reported. Here, we at first examined the ability of OxdA to catalyze the catalase reaction, which is one of the redox reactions. In order to investigate the catalase activity of OxdA, we measured the production of O_2_ by using an oxygen electrode. As a result, we observed production of O_2_ for the first time (S1A Fig). This finding suggests that OxdA catalyzes the catalase reaction. We also found that the heat-treated OxdA did not exhibit the catalase activity, strongly supporting that OxdA catalyzes the catalase reaction. Next, we carried out the catalase assaying of OxdA in reaction mixtures containing various concentrations of H_2_O_2_, and determined the kinetic parameters (Table 1). Moreover, we observed other redox activities (peroxidase and peroxygenase) of OxdA, and determined the kinetic parameters (Tables 2, 3 and 4). Our findings for the first time demonstrate that OxdA catalyzes three kinds of redox reactions among heme enzymes catalyzing non-redox reactions [51], while general heme enzymes catalyze redox reactions.

### Effects of the H320D mutation on redox activities

Here, we constructed OxdA(H320D) by the replacement of His320 with Asp to mimic the active site of CPO (S12B Fig). After the purification of OxdA(H320D), its redox activities (catalase, peroxidase and peroxygenase) were measured. As a result, OxdA(H320D) was found to exhibit three kinds of redox activities like OxdA(WT). Moreover, comparison of the determined kinetic parameters between OxdAs (WT and H320D) revealed that the *V*_*max*_ values of OxdA(H320D) were 36 (in the catalase reaction), 2.5 (in the peroxidase reaction with guaiacol), 9.7 (in the peroxidase reaction with ABTS), and 6.5 (in the peroxygenase reaction) times higher than those of OxdA(WT), respectively (Tables 1–4). It is apparent that the H320D mutation enhances all the redox activities. The higher redox activities of OxdA(H320D) would be caused by its onset potential (0.40 V vs. SHE), being higher than that of OxdA(WT) (0.28 V vs. SHE) (Fig 7). Furthermore, the *K*_m_ values for H_2_O_2_ (in the catalase reaction), guaiacol (in the peroxidase reaction with guaiacol), ABTS (in the peroxidase reaction with ABTS), and 1-MN (in the peroxygenase reaction) of OxdA(H320D) were equivalent to 130%, 3800%, 57% and 110% of those of OxdA(WT), respectively (Tables 1–4). These findings indicate that the H320D mutation significantly altered the affinity of OxdA for substrates.

**Fig 8:**
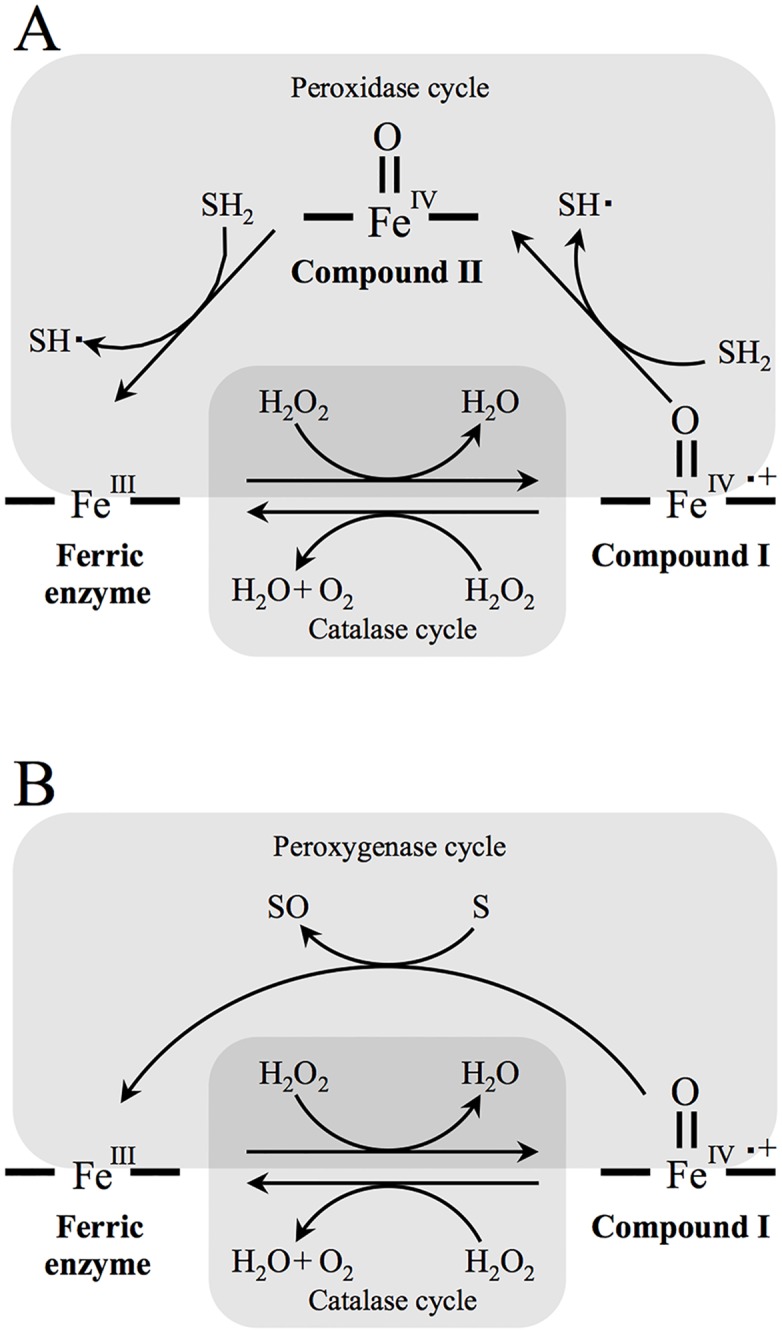
Proposed pathways of heme enzymes for the redox reactions. Peroxidase pathway (A), Peroxygenase pathway (B).

For all the redox reactions of OxdAs (WT and H320D) we measured, interesting findings suggesting a difference in the influence on these activities with a high concentration of H_2_O_2_ between OxdAs (WT and H320D) were obtained [S5 Fig, Figs 2A(a), 3A(a) and 4B]. Generally, a high concentration of H_2_O_2_ inhibits various enzymatic reactions due to its strong oxidizing power [49]. In the cases of OxdAs (WT and H320D), the catalase (S5 Fig) and peroxidase [Fig 2A(a)] activities (using guaiacol as a substrate) were inhibited by a high concentration of H_2_O_2_. For example, the catalase activity of OxdA(H320D) in the presence of 400 mM H_2_O_2_ (17 ± 1 units/mg) was about 3 times lower than that of 100 mM H_2_O_2_ (53 ± 2 units/mg). These inhibitions would be due to the above reason. These activities of OxdA(H320D) greatly decreased with high concentrations of H_2_O_2_ compared with those of OxdA(WT), indicating that the H320D mutation not only enhances the redox activities of OxdA but also would decrease its H_2_O_2_ tolerance. On the other hand, the peroxidase (using ABTS as a substrate) [Fig 3A(a)] and peroxygenase (Fig 4B) activities of OxdAs (WT and H320D) were significantly reduced by low concentrations (50–100 mM) of H_2_O_2_, although their catalase and peroxidase (using guaiacol as a substrate) activities were not reduced by the same concentrations of H_2_O_2_. For instance, the peroxidase activity of OxdA(H320D) using ABTS as a substrate in the presence of 100 mM H_2_O_2_ (9.2 ± 0.5 units/mg) was equivalent to 30% of that with 25 mM H_2_O_2_ (31 ± 1 units/mg), although the peroxidase activity of OxdA(H320D) using guaiacol as a substrate in the presence of 100 mM H_2_O_2_ (66 ± 6 units/mg) was equivalent to 140% of that with 25 mM H_2_O_2_ (48 ± 13 units/mg). Therefore, inhibition of the peroxidase (using ABTS as a substrate) and peroxygenase activities would be caused by competition between the catalase activity and these activities rather than H_2_O_2_ tolerance. During the catalase reaction, compound I [an enzyme intermediate, which contains an oxyferryl iron center and a second oxidizing equivalent stored as a radical (Fe(IV) = O^+•^) (formal oxidation state of +5)] [52] is produced and then consumed, resulting in a return to the native form (formal oxidation state +3) [52]. For the peroxidase reaction, compound I is produced and then consumed, yielding compound II [a second intermediate in which the radical is discharged leaving only the oxyferryl iron (formal oxidation state +4)] [52] (Fig 8A). As for the peroxygenase reaction, compound I is produced and then utilized to oxidize a substrate other than H_2_O_2_ (Fig 8B). During the peroxidase or peroxygenase reaction, the catalase reaction also proceeds because H_2_O_2_ contained in the reaction mixtures acts as a substrate for the catalase reaction. For the peroxidase (using ABTS as a substrate) and peroxygenase activity, respectively, the catalase reaction would proceed dominantly rather than the peroxidase or peroxygenase one in the presence of excess of H_2_O_2_ for the other substrate (ABTS or 1-MN). Namely, compound I would be utilized dominantly for the catalase reaction rather than the peroxidase or peroxygenase reaction. As a consequence, the peroxidase (using ABTS as a substrate) and peroxygenase activities would be inhibited. With a high concentration of H_2_O_2_, inhibition of the redox activities of OxdA(H320D) was greater than in the case of OxdA(WT) [Figs 3A(a) and 4B]. These results would be responsible for the higher catalase activity of OxdA(H320D) compared with that of OxdA(WT).

### Effects of the H320A mutation on redox activities

We used OxdA(H320A) to examine the effect of the charge of the 320th residue in the distal site of heme (S12C Fig). On measurement of its redox activities (catalase, peroxidase and peroxygenase), the catalase and peroxidase activities of OxdA(H320A) were found for the first time, while its peroxygenase activity was not detected. Furthermore, the kinetic parameters of its catalase and peroxidase activities were determined. The *V*_*max*_ value of its catalase activity was equivalent 140% of that of OxdA(WT) (Table 1), indicating that the H320A mutation did not markedly change its catalase activity. The catalase activity comparable with that of OxdA(WT) would be due to its onset potential (0.28 V vs. SHE), which is similar to that of OxdA(WT) (0.28V vs. SHE) (Fig 7). On the other hand, the *V*_*max*_ values of peroxidase activity using guaiacol as a substrate and peroxidase activity using ABTS as a substrate were equivalent to 6.0% and 38% of those of OxdA(WT), respectively (Tables 2 and 3), indicating that the H320A mutation decreases the peroxidase activity. The lower peroxidase activity of and lack of peroxygenase activity of OxdA(H320A) would be caused by removal of the charge of the 320th residue due to the H320A mutation. Furthermore, the *K*_m_ value for H_2_O_2_ (in the catalase reaction) of OxdA(H320A) was equivalent to 10% of that of OxdA(WT) (Table 1), indicating that the H320A mutation would significantly enhance its affinity for H_2_O_2_ due to its wide heme pocket. On the other hand, the *K*_m_ value for guaiacol (in the peroxidase reaction with guaiacol) and for ABTS (in the peroxidase reaction with ABTS) of OxdA(H320A) were equivalent to 3200% and 100% of those of OxdA(WT), respectively (Tables 2 and 3), indicating that the H320A mutation markedly decreased its affinity for guaiacol.

With a high concentration of H_2_O_2_, the inhibition of the peroxidase activity of OxdA(H320A) was greater than those of other OxdAs (WT and H320D) (Figs 2A and 3A). This would be caused by the lower *K*_m_ value for H_2_O_2_ of OxdA(H320A) than those of other OxdAs (WT and H320D). In the peroxidase reaction, the catalase reaction of OxdA(H320A) would proceed dominantly due to its higher affinity for H_2_O_2_ than those of other OxdAs (WT and H320D). Thus, the competition between its catalase activity and peroxidase activity in the peroxidase reaction would be greater than those of other OxdAs (WT and H320D).

### New reaction product in the peroxygenase reaction of OxdA(H320D)

In the reaction mixture with OxdA(WT), Russig’s blue was formed as a peroxygenase reaction product. On the other hand, in the reaction mixture with OxdA(H320D) instead of OxdA(WT), an increase in the absorbance of Russig’s blue (610 nm) was not detected at all. Alternatively, the color of the reaction mixture changed to a significantly different one with OxdA(WT) (Fig 5). Moreover, a characteristic absorption spectrum of the reaction mixture containing OxdA(H320D), which is different from that of the mixture containing OxdA(WT), was observed [Fig 6A(a)]. These findings suggest a new reaction product different from Russig’s blue. In order to identify the new product, the reaction mixture with OxdA(H320D) was analyzed by HPLC under the conditions given under “Materials and Methods.” As a result, the peak of the new reaction product was observed at 3.1 min in the UV-vis chromatogram. On the addition of inactivated OxdA(H320D) instead of OxdA(H320D), on the other hand, the peak was not observed (Fig 6B). Next, we purified the new reaction product by HPLC as described under “Materials and Methods,” and analyzed the purified product. In the UV-vis chromatogram, the peak of the purified new reaction product was observed at the same retention time (3.1 min) (Fig 6B), demonstrating that the new product present in the reaction mixture did not change into other compounds during the purification procedures. Based on the mass spectrum of the peak (Fig 6C), the molecular mass of the new reaction product is 174 in correspondence with the value of a 1 molecule oxygen adduct of 1-MN, suggesting that the new product is formed through hydroxylation. Furthermore, we measured NMR spectra of the purified new product (Fig 6D–6F), and identified it as 1-methoxy-2-naphthalenol, which has never been reported as a reaction product of the peroxygenase reaction. In the so-far known peroxygenase reaction with 1-MN as a substrate, the C-4 position of the naphthalene ring of 1-MN is hydroxylated to yield 4-methoxy-1-naphthalenol. Subsequently, two further peroxygenase reactions cause the condensation of two 4-methoxy-1-naphthalenols, resulting in the production of Russig’s blue [Fig 4A(a)]. In contrast, 1-methoxy-2-naphthalenol was produced through C-2 hydroxylation in the naphthalene ring of 1-MN [Fig 4A(b)], when OxdA(H320D) was used. However, the condensation of two 1-methoxy-2-naphthalenols through further peroxygenase reaction did not occur. The difference in the peroxygenase reaction product between OxdAs (WT and H320D) demonstrates that the H320D mutation significantly alters the hydroxylation site of 1-MN only by replacement of one amino acid residue. These findings would suggest that the direction of the naphthalene ring of 1-MN as to the heme iron may be quite different between OxdAs (WT and H320D). It is indicated that the His320 residue of OxdA is important for orientation of the substrate for catalysis of the redox reaction, and its mutation will cause a unique alteration of the reaction product. Moreover, we found no peroxygenase activity toward 1-MN in OxdA(H320A), which lacks acid-base behavior of the 320th residue in the aldoxime dehydratase reaction [32]. Based on the above findings and the peroxygenase activity of OxdA(WT) exhibiting acid-base behavior of His 320 in the aldoxime dehydratase reaction, and OxdA(H320D) possessing an Asp residue acting as an acid-base catalyst in redox reactions [41], the charge of the 320th residue would play a role in catalysis of the peroxygenase reaction for 1-MN.

In summary, our results for the first time demonstrate three kinds of redox catalysis of OxdA. Its H320D mutant showed higher redox activities and different affinities for substrates except for H_2_O_2_ from those of OxdA(WT). Also, its H320A mutant showed significantly higher affinity for H_2_O_2_ than that of OxdAs (WT and H320D). Furthermore, in the peroxygenase reaction, we observed a difference in the reaction product between the reactions of OxdAs (WT and H320D). These findings indicate that the His320 residue of OxdA is important for not only its the redox activities but also its substrate binding. Considering the extremely uncommon heme pocket of OxdA [30–32], there is a possibility that other unexpected reaction products could arise through the unique redox reactions of OxdA or its mutants. Further studies including structural analyses are required to elucidate the differences in the orientation of the substrate (1-MN) between OxdAs (WT and H320D).

## Supporting information

S1 Fig Assaying of the catalase reaction using OxdAs (WT, H320D and H320A). Time-dependent O_2_ production by OxdA(WT) (A), OxdA(H320D) (B) and OxdA(H320A) (C). The reaction mixture contained 10 mM H_2_O_2_ and 2 μM OxdA(WT), 1 μM OxdA(H320A), or 0.5 μM OxdA(H320D). The reactions were carried out under the “standard assay A” conditions as described under “Materials and Methods.” The reaction was initiated (arrow) by the addition of an OxdA (WT, H320D and H320A).
(PDF)

S2 Fig Absorption spectra of heat-treated OxdAs (WT, H320A and H320D). The absorption spectra of 3μM OxdA(WT) (A), OxdA(H320D) (B) and OxdA(H320D) (C) at pH 7.0 and 28°C before heat-treatment (thin line), or after heat-treatment (broken line) for 10 min at 98°C.
(PDF)

S3 Fig Hanes-Woolf plots of the catalase activities of OxdAs. The reactions were carried out under the “standard assay A” conditions as described under “Materials and Methods.” OxdA(WT) (A), OxdA(H320D) (B) and OxdA(H320A) (C).
(PDF)

S4 Fig Time-dependent increase in the absorbance at 470 nm of OxdAs. The reaction mixture contained 10 mM guaiacol and 25 mM H_2_O_2_. The reactions were carried out under the “standard assay B” conditions as described under “Materials and Methods.” OxdA(WT) (solid line), OxdA(H320D) (broken line) and OxdA(H320A) (thin line).
(PDF)

S5 Fig Catalase activities of OxdAs with a high concentration of H_2_O_2_. The reactions with high concentrations of H_2_O_2_ (100~400 mM) were carried out under the “standard assay A” conditions as described under “Materials and Methods.” For all data points, values are means ± mean error. OxdA(WT) (A), OxdA(H320D) (B) and OxdA(H320A) (C).
(PDF)

S6 Fig Hanes-Woolf plots of the peroxidase activities of OxdAs in the presence of guaiacol. The reactions were carried out under the “standard assay B” conditions as described under “Materials and Methods.” OxdA(WT) (A), OxdA(H320D) (B) and OxdA(H320A) (C).
(PDF)

S7 Fig Time-dependent increase in the absorbance at 430 nm of OxdAs. The reaction mixture contained 2.5 mM ABTS and 25 mM H_2_O_2_. The reactions were carried out under the “standard assay C” conditions as described under “Materials and Methods.” OxdA(WT) (solid line), OxdA(H320D) (broken line) and OxdA(H320A) (thin line).
(PDF)

S8 Fig Hanes-Woolf plots of the peroxidase activities of OxdAs in the presence of ABTS. The reactions were carried out under the “standard assay C” conditions as described under “Materials and Methods.” OxdA(WT) (A), OxdA(H320D) (B) and OxdA(H320A) (C).
(PDF)

S9 Fig Time-dependent increase in the absorbance at 610 nm of OxdA(WT) and OxdA(H320A). The reaction mixture contained 0.5 mM 1-MN and 5 mM H_2_O_2_. The reaction was carried out under the “standard assay D” conditions as described under “Materials and Methods.” 1 μM OxdA(WT) (solid line), 1 μM (thin line) and 10 μM (broken line) OxdA(H320A).
(PDF)

S10 Fig Hanes-Woolf plots of the peroxygenase activities of OxdA(WT) and OxdA(H320D). The reactions were carried out under the “standard assay D” conditions as described under “Materials and Methods.” OxdA(WT) (A) and OxdA(H320D) (B).
(PDF)

S11 Fig Time-dependent increase in the absorbance at 334 nm of OxdA(H320A). The reaction mixture contained 0.5 mM 1-MN and 5 mM H_2_O_2_. The reaction was carried out under the “standard assay D” conditions as described under “Materials and Methods.” 1 μM (thin line) and 10 μM (solid line) OxdA(H320A).
(PDF)

S12 Fig Schematic representation of the structural details of the active site of OxdAs. The structural details of OxdA(WT) (A), OxdA(H320D) (B), and OxdA(H320A) (C).
(PDF)
