# Correction: New function of aldoxime dehydratase: Redox catalysis and the formation of an expected product

**DOI:** 10.1371/journal.pone.0178974

**Published:** 2017-05-30

**Authors:** Masatoshi Yamada, Yoshiteru Hashimoto, Takuto Kumano, Seiya Tsujimura, Michihiko Kobayashi

In the title of this article, the word “expected” should be “unexpected.” The correct title is: New function of aldoxime dehydratase: Redox catalysis and the formation of an unexpected product. The correct citation is: Yamada M, Hashimoto Y, Kumano T, Tsujimura S, Kobayashi M (2017) New function of aldoxime dehydratase: Redox catalysis and the formation of an unexpected product. PLoS ONE 12(4): e0175846. https://doi.org/10.1371/journal.pone.0175846
