# Supplementary figures and images for: New function of aldoxime dehydratase: Redox catalysis and the formation of an expected product
Source: PLoS One. 2017 Apr 14;12(4):e0175846. doi: 10.1371/journal.pone.0175846 (PMC5391958; doi:10.1371/journal.pone.0175846)

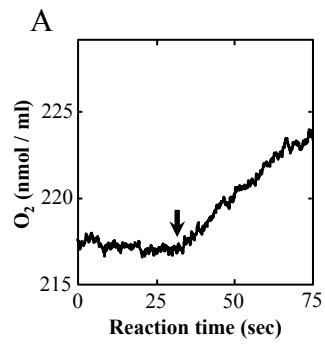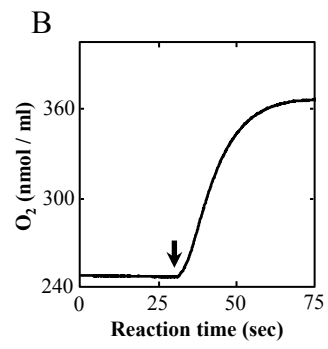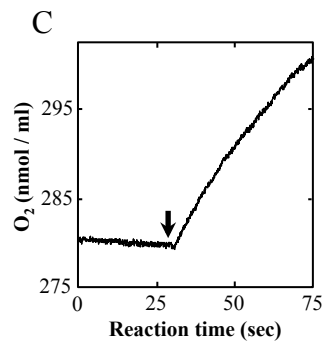

Supplement: S1 Fig — Time-dependent O2 production by OxdA(WT) (A), OxdA(H320D) (B) and OxdA(H320A) (C). The reaction mixture contained 10 mM H2O2 and 2 μM OxdA(WT), 1 μM OxdA(H320A), or 0.5 μM OxdA(H320D). The reactions were carried out under the “standard assay A” conditions as described under “Materials and Methods.” The reaction was initiated (arrow) by the addition of an OxdA (WT, H320D and H320A). (PDF) [file pone.0175846.s001.pdf]

**A**

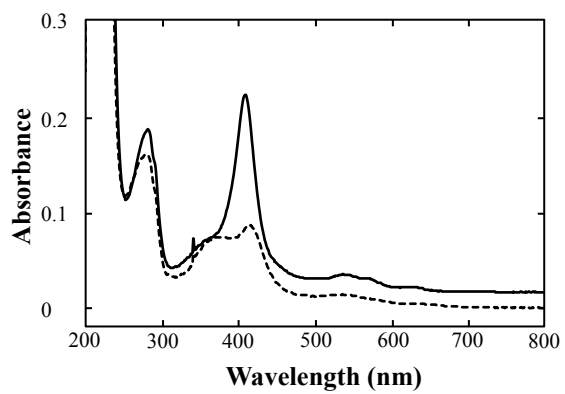

**B**

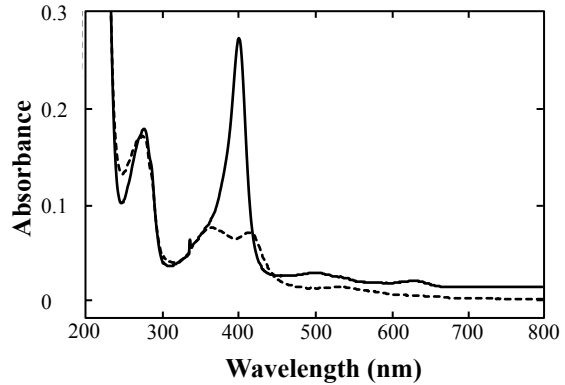

**C**

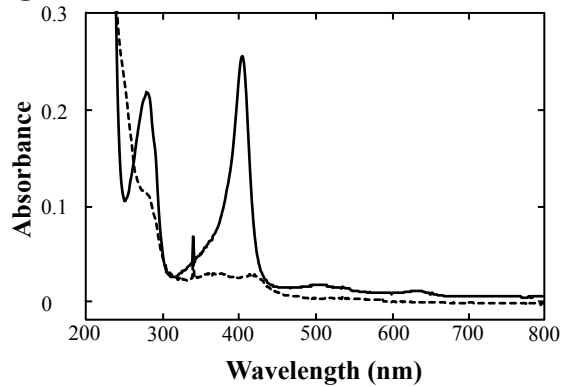

Supplement: S2 Fig — The absorption spectra of 3μM OxdA(WT) (A), OxdA(H320D) (B) and OxdA(H320D) (C) at pH 7.0 and 28°C before heat-treatment (thin line), or after heat-treatment (broken line) for 10 min at 98°C. (PDF) [file pone.0175846.s002.pdf]

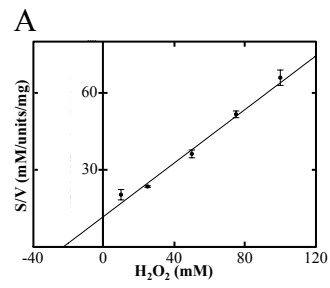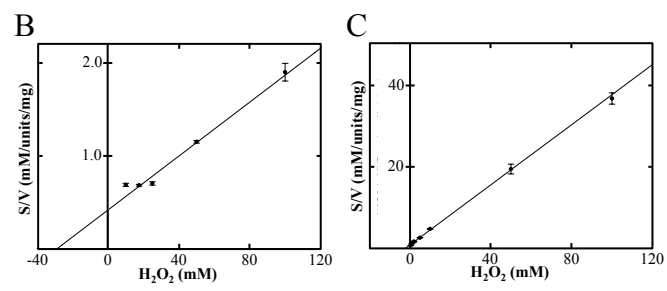

Supplement: S3 Fig — The reactions were carried out under the “standard assay A” conditions as described under “Materials and Methods.” OxdA(WT) (A), OxdA(H320D) (B) and OxdA(H320A) (C). (PDF) [file pone.0175846.s003.pdf]

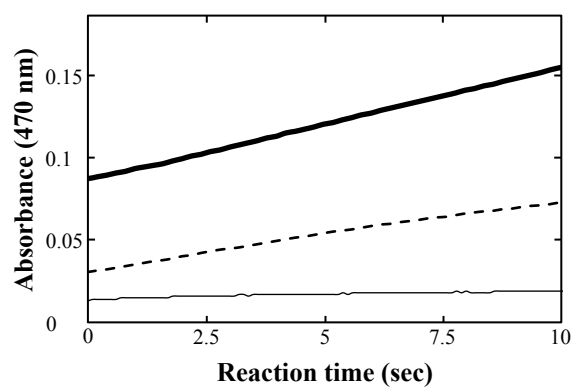

Supplement: S4 Fig — The reaction mixture contained 10 mM guaiacol and 25 mM H2O2. The reactions were carried out under the “standard assay B” conditions as described under “Materials and Methods.” OxdA(WT) (solid line), OxdA(H320D) (broken line) and OxdA(H320A) (thin line). (PDF) [file pone.0175846.s004.pdf]

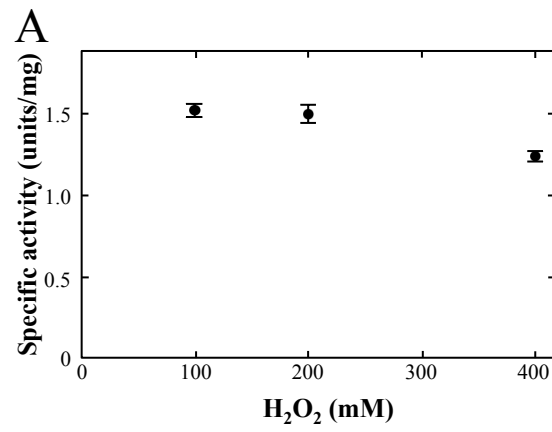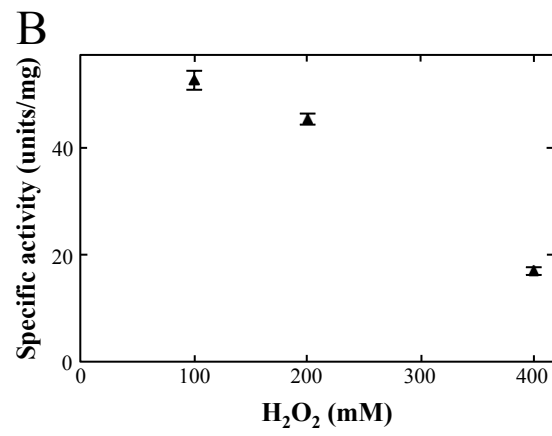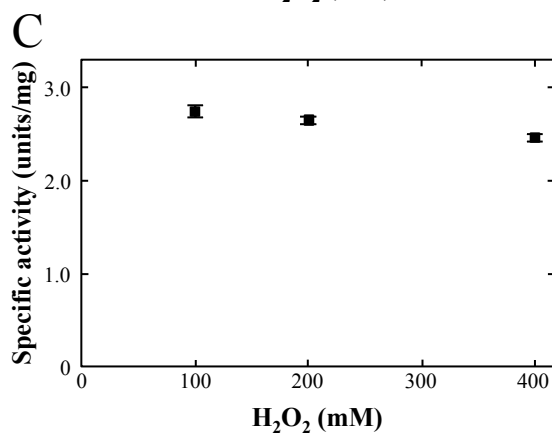

Supplement: S5 Fig — The reactions with high concentrations of H2O2 (100~400 mM) were carried out under the “standard assay A” conditions as described under “Materials and Methods.” For all data points, values are means ± mean error. OxdA(WT) (A), OxdA(H320D) (B) and OxdA(H320A) (C). (PDF) [file pone.0175846.s005.pdf]

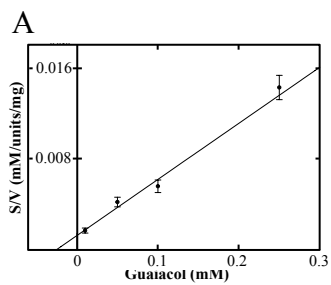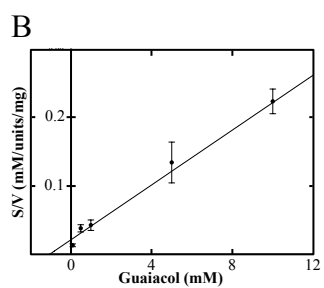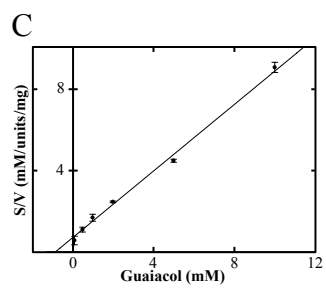

Supplement: S6 Fig — The reactions were carried out under the “standard assay B” conditions as described under “Materials and Methods.” OxdA(WT) (A), OxdA(H320D) (B) and OxdA(H320A) (C). (PDF) [file pone.0175846.s006.pdf]

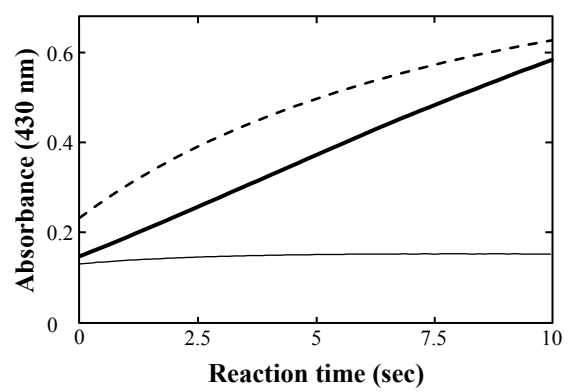

Supplement: S7 Fig — The reaction mixture contained 2.5 mM ABTS and 25 mM H2O2. The reactions were carried out under the “standard assay C” conditions as described under “Materials and Methods.” OxdA(WT) (solid line), OxdA(H320D) (broken line) and OxdA(H320A) (thin line). (PDF) [file pone.0175846.s007.pdf]

A

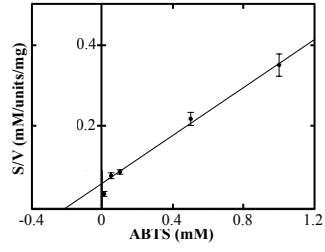

B

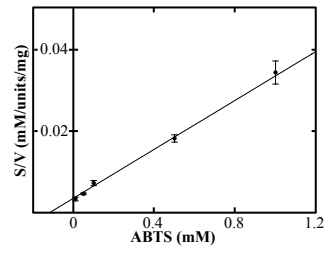

C

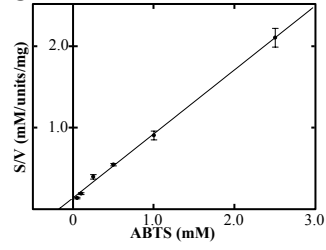

Supplement: S8 Fig — The reactions were carried out under the “standard assay C” conditions as described under “Materials and Methods.” OxdA(WT) (A), OxdA(H320D) (B) and OxdA(H320A) (C). (PDF) [file pone.0175846.s008.pdf]

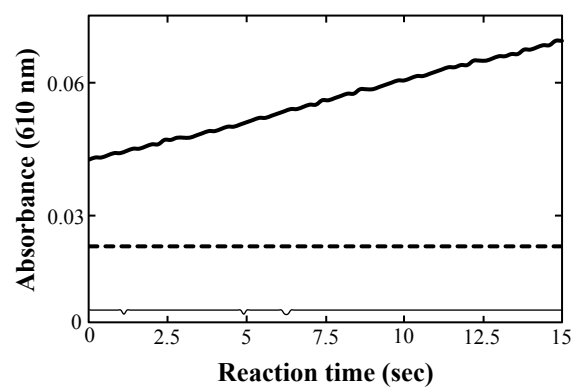

Supplement: S9 Fig — The reaction mixture contained 0.5 mM 1-MN and 5 mM H2O2. The reaction was carried out under the “standard assay D” conditions as described under “Materials and Methods.” 1 μM OxdA(WT) (solid line), 1 μM (thin line) and 10 μM (broken line) OxdA(H320A). (PDF) [file pone.0175846.s009.pdf]

A

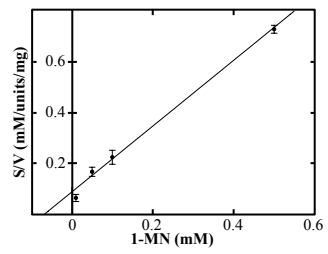

B

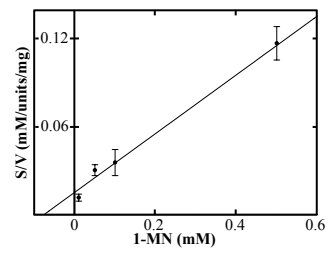

Supplement: S10 Fig — The reactions were carried out under the “standard assay D” conditions as described under “Materials and Methods.” OxdA(WT) (A) and OxdA(H320D) (B). (PDF) [file pone.0175846.s010.pdf]

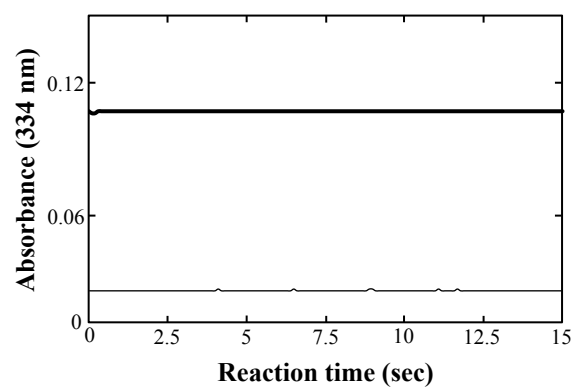

Supplement: S11 Fig — The reaction mixture contained 0.5 mM 1-MN and 5 mM H2O2. The reaction was carried out under the “standard assay D” conditions as described under “Materials and Methods.” 1 μM (thin line) and 10 μM (solid line) OxdA(H320A). (PDF) [file pone.0175846.s011.pdf]

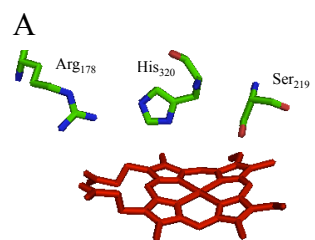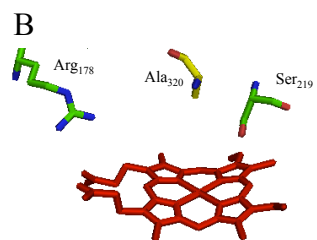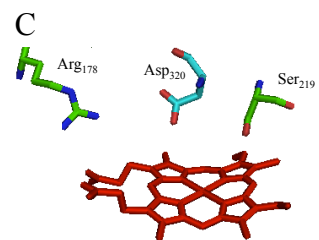

Supplement: S12 Fig — The structural details of OxdA(WT) (A), OxdA(H320D) (B), and OxdA(H320A) (C). (PDF) [file pone.0175846.s012.pdf]
